# Diastolic And Systolic Right Ventricular  Dysfunction Precedes Left Ventricular Dysfunction In Patients Paced From Right Ventricular Apex

**Published:** 2006-07-01

**Authors:** SK Dwivedi, Sandeep Bansal, Aniket Puri, MK Makharia, VS Narain, RK Saran, M Hasan, VK Puri

**Affiliations:** * Professor of Cardiology, Department of Cardiology, King George's Medical University, Lucknow, India.; † Senior Resident, Department of Cardiology, King George's Medical University, Lucknow, India.; ‡ Ex-Professor and Head, Department of Cardiology, King George's Medical University, Lucknow, India.; § Professor and Head, Department of Cardiology, King George's Medical University, Lucknow, India.

**Keywords:** diastolic dysfunction, systolic dysfunction, left ventricle, right ventricle, RV apical pacing

## Abstract

**Background:**

Cardiac dysfunction after right ventricular (RV) apical pacing is well known but its extent, time frame of appearance and individual effect on left ventricular (LV), RV systolic and diastolic parameters has not evaluated in a systematic fashion.

**Methods:**

Patients with symptomatic bradycardia and ACC-AHA Class I indication for permanent pacemaker implantation (PPI) were implanted a single chamber (VVI) pacemaker. They were followed prospectively by echocardiographic examination which was done at baseline, 1 week, 1 month and 6 months after implantation. Parameters observed were chamber dimensions (M-line), chamber volumes, cardiac output (modified Simpson's method), systolic functions (ejection fraction, pre-ejection period, ejection time and ratio) and diastolic functions( isovolumic relaxation time & deceleration time) of left and right heart.

**Results:**

Forty eight consecutive patients (mean age 65.6±11.8 yrs, 66.7% males, mean EF 61.82±10.36%) implanted a VVI pacemaker were enrolled in this study. The first significant change to appear in cardiac function after VVI pacing was in diastolic properties of RV as shown by increase in RV isovolumic relaxation time (IVRT) from 65.89±15.93 to 76.58±17.00 ms,(p<0.001) at 1week and RV deceleration time (DT) from 133.84±38.13 to 153.09±31.41 ms, (p=0.02) at 1 month. Increase in RV internal dimension (RVID) from 1.26±0.41 to 1.44±0.44, (p<0.05) was also noticed at 1 week. The LV diastolic parameters were significantly altered after 1 month with increase in LV-IVRT from 92.36±21.47 to 117.24±27.21ms, (p<0.001) and increase in LV DT from 147.56±31.84 to 189.27±28.49ms,(p<0.01). This was followed by LV systolic abnormality which appeared at 6 months with an increase in LVPEP from 100.33±14.43 to 118.41±21.34ms, (p<0.001) and increase in LVPEP/LVET ratio from 0.34±0.46 to 0.44±0.10, (p<0.001)]. The reduction in LV EF was manifested at 6 months falling from 61.82±10.36% to52.52±12.11%, (p<0.05) without any significant change in the resting cardiac output.

**Conclusion:**

The present study shows that dysfunction of right ventricle is the first abnormality that occurs in VVI paced patients, which manifests by 1 week followed by LV dysfunction which starts appearing by 1 month and the diastolic dysfunctions precede the systolic dysfunction in both ventricles.

## Introduction

Single chamber right ventricular apical pacing (VVI) continues to be the predominant pacing mode in the developing countries primarily due to economic constraints, despite the well-known advantages of dual chamber pacing. Pacemaker induced myocardial dysfunction has been recognized for a long time and the effect of single chamber right ventricular (RV) apical pacing (VVI) on left ventricular (LV) functions has been studied previously, yet  there remain some unexplored areas such as the chronology of events to occur [1-8]. Majority of studies have looked into LV dysfunction while effects on the right ventricle (RV) have not been studied thoroughly. Furthermore there is little data on the extent and the time frame in which these changes occur. We therefore, aimed to evaluate systolic and diastolic functions of LV and RV in consecutive patients undergoing VVI permanent pacemaker implantation (PPI) over a period of 6 months using trans-thoracic echocardiography.

## Material and Methods

All consecutive patients undergoing VVI pacemaker implantation having a class I indication according to AHA guidelines [9] were included in the study. A written informed consent was taken for inclusion in the study. Patients undergoing PPI implantations with other modes of pacing were excluded from the study.

### Clinical evaluation

A thorough clinical evaluation and resting 12-lead electrocardiogram (ECG) were done at admission, on day 7 after PPI and on follow up at 1 month and 6 months. Patients were discharged with a programmed pacing rate of 70/mt. At follow up visits, patients were evaluated for any evidence of a new onset dyspnea attributable to pacemaker syndrome, in the absence of any recognizable recent myocardial infarction, anaemia, respiratory illness and new LV ejection fraction of less than 50% [10,11]. The left ventricular function was evaluated by 2D echocardiography. At the end of 6 months follow up, a 24 hour ambulatory ECG was done to document the duration of paced and sinus rhythm in the previous 24 hours. A patient with paced rhythm of more than 90% of all ventricular complexes was considered to be on persistent pacing mode.

### Echocardiography protocol

Baseline echocardiographic evaluation was done within 24 hours of hospital admission with the temporary pacemaker rate being kept at 70/mt. In the event of intact atrio-ventricular (AV) conduction, echocardiography was done on right ventricular temporary pacing at a rate 10 beats higher than the  intrinsic sinus rate. Echocardiography was repeated at day 7, 1 month and 6 months after implantation.  Each measurement was taken in three consecutive paced beats not preceded by a sinus P wave and the average was considered as the representative value.

LV end diastolic and systolic volumes (LVEDV and LVESV) were calculated using modified Simpson's method. Left ventricular internal diameter in diastole (LVIDd) and systole (LVIDs) and fractional shortening (FS) were measured in parasternal long axis view using the M-mode. The right ventricular internal diameter in diastole (RVIDd) and systole (RVIDs)  were taken in the parasternal long axis view and their mean was taken as the RVID. Left atrial size was measured in systole (LAs) and diastole (LAd) on M-line in parasternal long axis view. Left sided systolic time intervals; LV pre ejection period (LVPEP) and LV ejection time (LVET) were calculated using Doppler aortic flow. Right sided systolic time intervals, RV pre ejection period (RVPEP), and RV ejection time (RVET) were calculated  using Doppler pulmonary flow. The LV diastolic parameters were calculated in apical 5-chamber view keeping the Doppler cursor at a point where both aortic outflow and mitral inflow could be obtained. The LV isovolumic relaxation time (LV-IVRT) was taken as a period from end of aortic flow to beginning of mitral inflow. The acceleration and deceleration time of mitral inflow (MV-AT and MV- DT) were measured from the onset of the mitral inflow to its peak and from peak to return to baseline respectively with the Doppler cursor placed just beyond the mitral annulus in apical four-chamber view. The RV isovolumic relaxation time (RV-IVRT) was calculated using two views. First, the RV electromechanical systole was calculated in parasternal short axis view using Doppler pulmonary flow. This was taken as the period from Q-wave on ECG to the completion of pulmonary flow. An average of three such readings was taken. Then the tricupid valve Doppler inflow tracing was taken in apical four chamber view in the manner similar to mitral inflow. The RV electromechanical systole was marked on this. This point was taken as beginning of the RV diastole and the period from this point to the beginning of tricuspid inflow was taken as RV-IVRT. The tricuspid acceleration and deceleration time (RV-AT and RV-DT) were measured in a manner similar to mitral AT and DT. The systemic and pulmonary cardiac outputs were calculated using velocity time integral (VTI) method across aortic and pulmonary Doppler flow respectively. Colour Doppler was used to conform and quantify AV valvar regurgitation, using area of the regurgitant jet relative to the size of LA [10,12].

### Statistical analysis

Student 't' test was used for statistical evaluation. The paired 't' test was used to compare the baseline values with the values at day 7, 1 month and 6 months respectively. A 'p' value of <0.05 was considered to be significant. Parameters compared were LVIDd, LVIDs, LVEDV, LVESV, RVID, LVEF, LVFS, LVPEP, LVET, RVPEP, RVET, LAd, LAs, systemic and pulmonic cardiac output, LV-IVRT, LV- AT& DT, RV-IVRT, RV- AT & DT. The effect of confounding parameters as age (< 50 versus > 50 years), sex (male versus female), indication (complete heart block versus Sick Sinus Syndrome), LV dysfunction (LVEF < 50% versus > 50%) and the requirement of pacing  (intermittent versus regular) were also studied using two way 't' test.

## Results

Forty-eight patients underwent VVI pacing. Three of the 48 patients had poor echo window and were  excluded from the final analysis. Over the 6 months follow up, 2 patients died. One patient had a haemorrhagic stroke and the other having documented coronary artery disease died suddenly. Two patients were lost to follow up after discharge but postal inquiries showed that they had no fresh symptoms. Data could therefore be obtained for all 45 patients at baseline and day 7, in 44 patients at 1 month and 41 patients at 6 months.

Baseline characteristics of the patients are given in Table 1. The study population consisted of 66.7% males with a mean age of  65.6±11.8 years (range 35-90years). Thirty four (75.6%) patients had complete heart block (CHB) and remaining (24.4%) had sick sinus syndrome (SSS). The mean LVEF of the group was 61.8±10.4%. Seven patients had LVEF of < 50% with 5 having dilated cardiomyopathy with normal coronaries and 2 having coronary artery disease, 1 patient had complete heart block secondary to inferior wall MI  persisting beyond 3 weeks and 5  patients had co-existing coronary artery disease (CAD) of varying duration.

Syncope did not recur in any patients over the 6 month period. Eight patients who had presented on admission with dyspnoea experienced initial relief with drug therapy but dyspnoea worsened in 4 of these patients. Over the 6 months follow up period new onset dyspnoea occurred in 7 patients. In 4 patients, it was considered as a part of pacemaker syndrome while in 3 it was attributed to progressive LV dysfunction. New onset persistent atrial fibrillation developed in 9 patients, it was noted at the end of 1 month in 2 patients and at 6 months in remaining 7 patients. Nine patients were adjudged to be having intermittent pacing showing <90% paced complexes on 24 hours ambulatory ECG monitoring at 6 months.

Cardiac dimensions, volumes, systolic functions and systolic time intervals are shown in (Table 2). The LVEDV and LVESV increased significantly by 6 months over their corresponding baseline values (p<0.05). The right ventricular dimension (RVID) showed an earlier increase from baseline (p<0.05) at day 7 and progressively increased in the 6 months follow up period. Significant increase in left atrial diastolic diameter (LAd) was observed at 6 months (p<0.05). The left atrial systolic diameter (LAs) also increased but not statistically significant (p>0.05). The LVEF decreased progressively from baseline (61.82 ± 10.36%)  and  was statistically significant at 6 months (52.52 ± 12.11%, p<0.05). Systemic and pulmonic resting cardiac output did not change significantly over the six months follow up period. The LVPEP increased significantly by 6 months (p<0.001) while LVET decreased over the same period (p<0.05). The RVEP increased significantly by 1 month (p<0.001) and continued to increase progressively thereafter. The RVET decreased significantly by 6 months (p<0.02).

Among the diastolic parameters (Table 3), LV-IVRT increased significantly at 1 month (p<0.001) and continued to increase progressively till 6 months. The mitral valve DT also increased similarly becoming statistically significant by 1 month (p<0.01). The right ventricular parameters showed a change much earlier than corresponding LV parameters. RV-IVRT showed a significant increase as early as day 7 (p<0.01) and continued to do so during the 6 month follow up period. The RV-DT also showed a significant increase by 1 month (p<0.02).  The ventricular filling acceleration time (AT) did not change significantly during the entire period in either of the two ventricles. New valvar regurgitation was seen in 7 patients. Of these, 4 had mild mitral regurgitation (MR) while 3 had mild tricuspid regurgitation (TR). Two of these patients had both MR and TR co-existent. One patient had pre-existing MR and TR, which worsened after pacemaker implantation.

The changes observed were largely uniform and independent of age, sex, indication for pacing and pre-existing LV dysfunction. Only persistent pacing, as identified by >90% paced beats  in a 24 hour holter, was associated with a significantly higher decrease in LVEF (p<0.02) in persistently paced group. This group also showed a higher LVESV (p<0.005), greater RV dysfunction as suggested by higher RVPEP/RVET ratio (p=0.024), and a tendency to have a larger LAd (p=0.063).

## Discussion

In the present study, the first significant change to appear in myocardial functions following VVI pacing was in diastolic properties of RV. This was manifest by an increase in RV-IVRT at one week, followed by an increase in RV dimensions and the appearance of RV systolic abnormality i.e. increased RV-PEP and RV-PEP/RV-ET ratio at one month. The LV diastolic parameters were significantly altered at one month, which were manifest by an increase in IVRT and DT, followed by LV systolic dysfunction which appeared at 6 months with an increase in LV-PEP and LV-PEP/LV-ET ratio. The reduction in LVEF also manifest at 6 months without change in the resting cardiac output.

Our findings suggest two inter-related sequence of events. Firstly, it appears that changes in cardiac haemodynamics start appearing as early as one-week after VVI pacing, which are subtle and are limited to right ventricle only. Left ventricular dysfunction occurs later and this follows RV dysfunction, as reported in previous studies [4,6-8]. Secondly, diastolic abnormalities are first to appear which are followed by appearance of systolic abnormalities of the respective ventricles. Our study is unique in highlighting the sequence in change in the LV and RV diastolic and systolic function in a serial fashion in VVI paced patients.

There is often a debate whether LV dysfunction after long term ventricular pacing is real or is dependent on pre-existing disease. Mohan et al4 in their study found no significant difference in resting LVEF in long term follow up of AAI and VVI paced patients of sinus node dysfunction without structural heart disease. Similarly, Anderson et al [6], in their study of sick sinus syndrome patients, found no difference in incidence of clinical congestive heart failure or LV dimensions between AAI and VVI pacing modes on  long term follow up. On the other hand, Faerestrand et al [13] found an increase in end diastolic volume in VVI paced patients after 3 months. Likewise, Fehrsson et al7 found greater resting LV volumes and lesser EF in patients paced on VVI rather than with physiological pacing mode. In most of these studies, stress has been on systolic function of LV after long term pacing while few have looked into the serial follow up of these patients. Data  regarding the diastolic function is conflicting. LeClercq et al [14] in a study of 11 patients found that filling rates improved with AAI pacing as compared to DDD pacing, and similar Doppler data was provided in another report by Rosenqvist et al [15]. However data from Vardas et al [16] found no difference in the the diastolic Doppler indices between AAI and DDD modes of pacing. Moreover, the systolic functions of right ventricle as well as diastolic functions of both the ventricles have not been studied comprehensively.

The true mechanism of cardiac dysfunction is still unclear. It has been shown that pacing from RV apex induces an electrical sequence of depolarisation, which results in reduced stroke output of both the ventricles [`7,^18^]. RV apical pacing induces a left bundle branch block pattern and studies have shown that alteration in normal activation sequence adversely affects LV function as demonstrated by decrease in the global ejection fraction [19]. This is supported by finding that near normal activation sequence as seen in RV outflow tract pacing results in a narrowed QRS and improved cardiac output [20]. Karpawich and Mital, in  a report, have also shown a normalized LV function with septal pacing despite a loss in atrial contribution as compared to atrial pacing [21]. Resultant chronic reduction in stroke output may mediate a compensatory mechanisms leading to cardiac chamber enlargement in an effort to maintain requisite stroke volume by Starling's law. There is evidence that delayed depolarisation of the LV can result in augmented intra-myocardial pressure in the septum and affect myocardial perfusion. Kolletis et al [22] have shown that atrial or AV sequential pacing does not alter coronary flow reserve while ventricular pacing decrease resting coronary flow velocity in some patients. Furthermore, long term RV apical pacing also results in myocardial perfusion defects on nuclear studies [23]. The magnitude of these defects is shown to be proportional to the duration of pacing and  are associated with apical wall motion abnormalities and impaired global LV function [23].

Beside this, animal experimental studies have shown myofibrillar disarray [24], redistribution of myocardial fibre strain and blood flow [25] and adverse histopathological changes [26] on long term pacing in canine hearts which may also contribute to systolic dysfunction on RV apical pacing. Since repolarization sequence is determined by depolarization sequence, myocardial relaxation is also likely to be non-homogeneous and diastolic abnormalities are likely to appear. The diastolic filling parameters on radionuclide left ventriculography, such as time to peak filling rate and negative dp/dt are significantly altered in ventricular paced animal models [27] as well as in human hearts on atrioventricular pacing [28].

However, these mechanisms are not enough to explain significant reduction in systolic performance of the left ventricle as adjudged by gross reductions of the LVEF on long term pacing. It is hypothesized that left ventricular diastolic and systolic functions are deranged in many conditions secondary to involvement of right ventricle. Isolated right ventricular infarction, atrial septal defect with right sided chronic volume over load, cor pulmonale and chronic obstructive lung disease result in altered LV diastolic as well as systolic functions secondary to RV dysfunction [29-32]. LV dysfunction in paced patients may be a consequence of RV dysfunction which occurs earlier as is shown in the present study. However, more studies are required to have a firm explanation to this hypothesis.

### Study Limitations

Echocardiographic evaluation of RV size may not be a reliable and reproducible method with inherent problems of inter and intra observer variability. However, since every patient was serving as their own control, and a change in the parameters could be attributed to permanent pacing. The study was designed to include consecutive patients undergoing single chamber VVI pacing and those having a baseline ejection fraction of less than 50% were also included, however in the final analysis on comparison with patients having ejection fraction greater than 50% there was no difference in the observed parameters. A critically timed atrial contraction can change the filling characteristics of ventricle and alter the measured diastolic and systolic parameters. Though, we chose to evaluate only paced complexes, it may not have completely removed the impact of atrial contribution to ventricular filling during the intervening sinus rhythm.

In conclusion, the present study shows that right ventricular apical pacing has a major effect on systolic and diastolic ventricular functions. On serial evaluation, right ventricle dysfunction is the first abnormality that occurs in right ventricular apical paced patients, followed by LV dysfunction which appears later and on both sides, diastolic dysfunction precede the systolic dysfunction.

## Figures and Tables

**Table 1:**
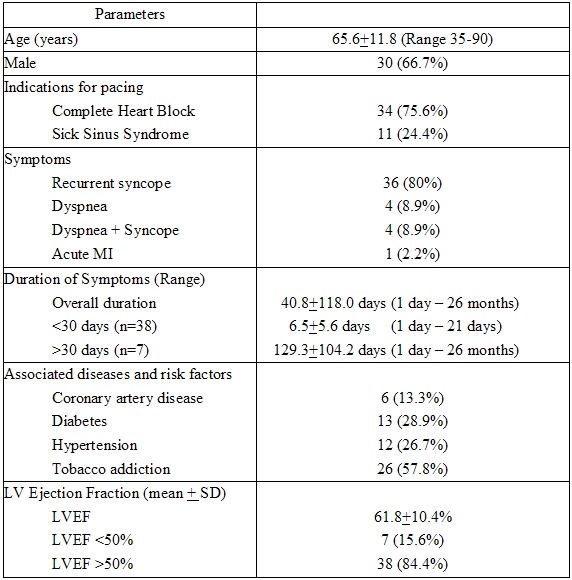
Baseline Clinical Characteristics of Study Patients (n=45)

( LVEF= Left Ventricular ejection fraction)

**Table 2:**
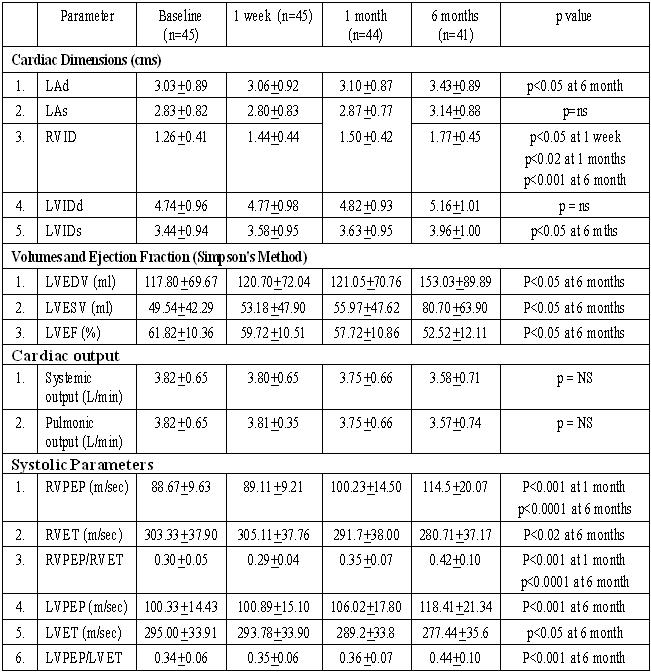
Changes in Cardiac Dimensions

(LAd = Left atrial diameter in diastole, LAs = Left atrial diameter in systole, RVID = Right ventricular internal diameter, LVIDd = End diastolic left ventricular internal diameter, LVIDs = End systolic left ventricular internal diameter, LVEDV = Left ventricular end diastolic volume, LVESV = Left ventricular end systolic volume, RVPEP = Right ventricular pre-ejection period; RVET = Right ventricular ejection time; LVPEP = Left ventricular pre ejection period, LVET = Left ventricular ejection time)

**Table 3:**
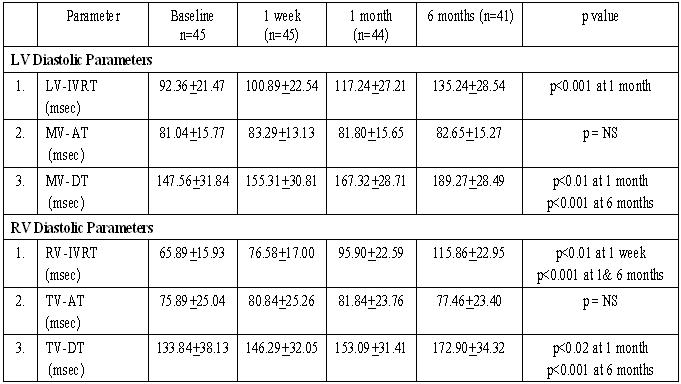
Changes in Diastolic Parameters

(LV-IVRT = Left ventricular isovolumic relaxation time, MV-AT = Mitral valve acceleration time, MV-DT = Mitral valve deceleration time, RV-IVRT = Right ventricular isovolumic relaxation time, TV-AT = Tricuspid valve acceleration time, TV-DT = Tricuspid valve deceleration time)

## Glossary of Abbreviations

PPI: permanent pacemaker implantation
VVI: Single chamber Right ventricular apical pacemaker implantation
LV: Left ventricular
RV: Right ventricular
LAd: Left atrial diameter in diastole
LAs: Left atrial diameter in systole
LVIDd: End diastolic left ventricular internal diameter
LVIDs: End systolic left ventricular internal diameter
LVEDV: Left ventricular end diastolic volume
LVESV: Left ventricular end systolic volume
RVIDd: End diastolic right ventricular internal diameter
RVIDs: End systolic right ventricular internal diameter
RVID: Mean Right ventricular internal diameter
LVEF: Left ventricular ejection fraction
LVFS: Left ventricular fractional shortening
LVPEP: Left ventricular pre ejection period
LVET: Left ventricular ejection time
RVPEP: Right ventricular pre-ejection period
RVET: Right ventricular ejection time
LV-IVRT: LV isovolumic relaxation time
LV- AT: mitral flow acceleration time
LV- DT: mitral flow deceleration time
RV-IVRT: RV isovolumic relaxation time
RV- AT: tricuspid flow acceleration time
RV- DT: tricuspid flow deceleration time
CAD: coronary artery disease
CHB: Complete Heart Block
AHA: American Heart Association
